# Key cellular subpopulations and mechanisms of propranolol in infantile hemangioma: insights from single-cell omics

**DOI:** 10.3389/fmed.2026.1795094

**Published:** 2026-05-13

**Authors:** Meng Chen, Jun Liu, Jiaxin Meng, Qiang Chen

**Affiliations:** 1 Department of Pediatric Surgery, Chongqing University Three Gorges Hospital, Chongqing, China; 2 Department of Pharmacy, Chongqing University Three Gorges Hospital, Chongqing, China; 3 Department of Anesthesiology, Chongqing Medical University, Chongqing, China

**Keywords:** cellular heterogeneity, infantile hemangioma, mechanism, propranolol, single-cell RNA sequencing

## Abstract

Infantile hemangioma (IH) is the most common benign vascular tumor in infancy, characterized by rapid proliferation followed by spontaneous involution. Despite propranolol being established as first-line therapy, the cellular basis of this biphasic behavior and the mechanisms underlying propranolol efficacy have remained incompletely understood. Single-cell RNA sequencing (scRNA-seq) has recently enabled high-resolution dissection of IH tissue composition, revealing a previously uncharacterized cellular heterogeneity that advances our understanding of both disease pathogenesis and drug action. Here we systematically review these advances. Within the vascular compartment, APLN-positive endothelial cells with tip cell characteristics and CENPF-positive proliferative pericytes are identified as IH-specific subpopulations enriched in the proliferating phase and suppressed by propranolol treatment. CD146-positive mural cells, which constitute the predominant cell population in IH, exhibit a dynamic proangiogenic-to-adipogenic transition that may underlie the spontaneous proliferation-to-involution switch. Beyond vascular cells, macrophages, mast cells, and telocytes within the stromal microenvironment contribute to lesion progression and modulate drug response. These findings support a model in which IH progression is governed by dynamic shifts in dominant cellular subpopulations rather than endothelial hyperproliferation alone, and suggest how propranolol may act across multiple cell types. These insights may inform efforts to predict treatment response, assess rebound risk, and refine therapeutic strategies for IH.

## 1 Introduction

Infantile hemangioma (IH) represents the most prevalent benign vascular tumor during infancy, affecting approximately 4%–10% of infants, with higher incidence among female infants and those born prematurely or with low birth weight (1–3). The hallmark of IH is its distinctive biphasic lifecycle: rapid proliferation during the first weeks to months of life, followed by gradual spontaneous involution typically beginning around 1 year of age, with most lesions resolving completely or partially over subsequent years. Although the majority of affected children have favorable outcomes, approximately 10%–15% of cases develop serious complications including ulceration, hemorrhage, airway obstruction, or visual impairment due to lesion location or size, necessitating early intervention (2–4).

The serendipitous discovery by Léauté-Labrèze et al. (5) in 2008 that propranolol exhibits substantial efficacy against IH established it as the first-line treatment for high-risk IH. The landmark randomized controlled trial by Léauté-Labrèze et al. demonstrated that oral propranolol at 3 mg/kg/day for 6 months achieved complete or near-complete resolution in 60% of patients at week 24, compared with 4% in the placebo group, with clinically meaningful improvement in 88% of patients by week 5 (6). Observational studies have reported overall improvement rates of 96%–98%, likely reflecting broader eligibility criteria and longer follow-up durations (7). However, the precise mechanisms underlying propranolol’s therapeutic effects remain incompletely elucidated. In clinical practice, the most commonly reported adverse effects are mild and reversible, including gastrointestinal disturbance, sleep disturbance, and acrocyanosis; serious effects such as hypoglycemia, bradycardia, and bronchospasm occur infrequently, though infants with risk factors warrant heightened monitoring during initiation (8). Shah et al. reported that rebound growth was significantly more likely when treatment duration was less than 9 months, supporting current guideline recommendations for extended treatment (9).

While propranolol has become first-line therapy for high-risk IH, the management of IH remains multifaceted. Topical timolol maleate is used for superficial, thin lesions, and meta-analyses confirm comparable efficacy for early superficial IH (10). Pulsed-dye laser (PDL) and other laser modalities are employed for residual telangiectasia, ulceration, or cosmetically sensitive lesions. Surgical excision is reserved for lesions causing functional impairment, pedunculated lesions amenable to simple resection, or residual fibrofatty tissue following involution. Historically, systemic corticosteroids and vincristine were used in refractory or complex cases (11). Not all IH lesions require active treatment–small, asymptomatic lesions in non-critical locations may be managed with watchful waiting, given the natural history of spontaneous involution (12). Certain anatomical locations and clinical presentations create particular management challenges. Periocular IH, even when small, may induce amblyopia through ptosis, astigmatism, or direct visual axis obstruction, necessitating urgent intervention. Subglottic IH poses life-threatening airway compromise and requires multidisciplinary otolaryngological input. Ulcerated IH, occurring in approximately 15%–25% of treated lesions, causes pain, bleeding, secondary infection, and scarring. Segmental, large, or PHACE syndrome-associated IH present additional diagnostic and treatment complexity, often requiring multidisciplinary evaluation and careful risk stratification before initiating systemic therapy. These scenarios underscore that IH management is not a “one-size-fits-all” medical prescription but requires individualized, multidisciplinary strategies.

The complexity and heterogeneity of these clinical scenarios underscore the importance of understanding the underlying disease mechanisms. Two questions remain central to the field: the cellular mechanisms governing the transition from proliferation to involution are not well defined, and the precise cellular targets of propranolol have yet to be fully characterized. Conventional bulk transcriptomic approaches have characterized IH at the population level but lack the resolution to distinguish functionally distinct cell subsets within the tumor. The advent of scRNA-seq has begun to address this limitation, revealing cellular heterogeneity that was previously invisible and identifying subpopulations with distinct roles in disease progression and drug response. It should be noted that the number of dedicated scRNA-seq studies in IH remains limited, and the findings reviewed here are largely derived from a small number of datasets. Broader and more longitudinal single-cell studies are needed before definitive conclusions can be drawn. With this caveat in mind, this review examines what single-cell omics studies have revealed about the cellular composition of IH and the cellular basis of propranolol action.

## 2 Cellular landscape of IH: from bulk to single-cell resolution

The traditional view that IH results primarily from endothelial cell hyperproliferation has been refined by accumulating evidence demonstrating that IH development and progression involve coordinated interactions among multiple cell types. scRNA-seq has since allowed more granular characterization of IH tissue composition, identifying cellular subpopulations with distinct functional roles in disease progression and drug response.

### 2.1 Endothelial cell lineage and key subpopulations

The pathological hallmark of proliferating IH is endothelial cell hyperproliferation, forming densely packed and irregularly arranged vascular luminal structures. Glucose transporter 1 (GLUT1) serves as a specific marker for IH endothelial cells, distinguishing IH from other vascular tumors and malformations (13). scRNA-seq studies have revealed significant heterogeneity within the endothelial lineage, with different subpopulations potentially playing distinct roles in lesion expansion and treatment response.

Chen et al. (14) identified through comprehensive scRNA-seq analysis that APLN-expressing endothelial cell subpopulations are significantly enriched in proliferating IH and are markedly reduced following propranolol treatment. Further investigation confirmed that this subpopulation promotes endothelial cell proliferation and migration through the Apelin/APLNR (APJ) signaling axis (14). Clinical sample analysis demonstrated that serum apelin levels correlate positively with lesion area in superficial IH, with significantly higher levels in proliferating-phase patients compared to healthy controls (15). These findings suggest that APLN-positive endothelial cells may constitute the cellular basis for the “high angiogenic state” in IH, while apelin may serve as a serum biomarker for disease staging and treatment monitoring. Independent studies have corroborated the importance of the Apelin/APLNR axis in IH, with researchers proposing APLNR as a specific marker and potential therapeutic target for IH-associated angiogenesis (16). A contemporaneous review by Xiang et al. further contextualized these findings within the broader landscape of IH angiogenesis, demonstrating that disrupted vascular equilibrium at the lesion site – driven by interactions among endothelial cells, immune cells, and stromal components – underlies the aberrant vessel network formation characteristic of IH, and that propranolol may exert its effects across this multicellular axis (17).

### 2.2 Pericyte and mural cell lineages

Pericytes enwrap microvascular endothelial cells and participate in vascular stability regulation and remodeling. While previous research focused primarily on the scaffolding function of pericytes, recent studies indicate that pericytes themselves exhibit functional heterogeneity, with certain subpopulations potentially directly participating in lesion expansion or involution. IH-derived pericytes (Hem-pericytes) differ from retinal pericytes in their capacity to accelerate the proliferation and migration of endothelial colony-forming cells (ECFCs) in co-culture, and themselves overexpress VEGF-A and exhibit dysregulated angiopoietin-1, collectively demonstrating pronounced proangiogenic properties (18).

Single-cell RNA sequencing analysis identified a distinct CENPF-expressing pericyte subtype that is closely associated with cell cycle and mitotic activity and is uniquely enriched in IH relative to normal skin tissue (14). CENPF is a centromere-associated protein predominantly expressed during the G2/M phase; Ki-67 co-staining and immunofluorescence validation confirmed that CENPF-positive perivascular cells represent a highly proliferative pericyte population that decreases significantly following propranolol treatment (14, 19). This finding indicates that pericytes contribute actively to lesion expansion, extending their role beyond that of passive vascular stabilizers.

Chen et al. (20) employed CD146 magnetic bead sorting to isolate a population of mural-like cells (hemangioma mural cells, HemMCs) from IH tissue that exhibited the most significant changes during the proliferation-to-involution transition. CD146-positive HemMCs possess mesenchymal stem cell characteristics, with transcriptomic sequencing revealing enhanced proangiogenic pathway activation. Functional studies demonstrated that HemMCs promote tube formation by human umbilical vein endothelial cells (HUVECs) *in vitro*; *in vivo*, combined HemMC and HUVEC implantation generates GLUT1-positive IH-like vascular structures that subsequently undergo spontaneous transformation into adipose tissue. This finding suggests that CD146-positive HemMCs may represent the key cellular population driving the unique “proliferation followed by involution” disease course of IH, with their dual differentiation potential (proangiogenic plus adipogenic) closely matching the natural history of IH. Unlike CD133-positive hemangioma stem cells (HemSCs), which constitute only 0.2% of IH cells, CD146-positive HemMCs are more abundant and therefore more suitable as cellular models for studying IH progression and drug response (20).

### 2.3 Immune cells and stromal microenvironment

The role of the immune microenvironment in IH progression has received increasing research attention. Macrophages represent the predominant immune cell component in IH tissue and can exhibit different polarization states with corresponding functions (21). Studies have shown that macrophages participate in lesion progression by promoting endothelial differentiation and inhibiting HemSC adipogenesis (21). Exosomes derived from M2-polarized macrophages can reduce HemSC sensitivity to propranolol via miR-27a-3p (22), suggesting that the immune microenvironment may modulate drug response. Mast cells participate throughout the “proliferation-involution” process. During the proliferating phase, mast cells accumulate within IH stroma and release a range of proangiogenic mediators including stem cell factor (SCF), VEGF, and basic FGF, which sustain endothelial proliferation and recruitment of progenitor cells. As lesions involute, mast cell density declines and their secretory profile shifts, with their secretory role in tissue remodeling during involution remains under investigation (23). Whether propranolol directly modulates mast cell number or function in IH remains to be established (23, 24).

Additionally, telocytes (TCs), a specialized stromal cell population, can participate in tissue microenvironment regulation through cellular contact and paracrine signaling. Research has reported the presence of AQP1-expressing TC layers surrounding IH vessels, forming spatial relationship networks with CD146-positive mural cells, α-SMA-positive cells, and CD31-positive endothelial cells (25). This anatomical positioning places telocytes at the interface between perivascular and endothelial compartments, consistent with a role in coordinating angiogenic signals across cell types. Aquaporin-1 expression in telocytes suggests a role in local fluid homeostasis (25). Although direct evidence for propranolol’s effects on telocyte biology in IH is currently lacking, the spatial proximity of AQP1-positive TCs to CD146-positive HemMCs–the population most dynamically altered by treatment–makes telocytes a candidate population for future investigation (25).

Recent studies have highlighted the critical role of non-coding RNAs (ncRNAs) in IH pathogenesis, particularly microRNAs (miRNAs), long non-coding RNAs (lncRNAs), and circular RNAs (circRNAs). These ncRNAs regulate key cellular processes including proliferation, apoptosis, migration, and angiogenesis. Several miRNAs have been reported as important regulators in IH, among which miR-206, miR-424, and miR-203a-3p have been implicated in modulating key angiogenic and pro-survival signaling pathways relevant to disease progression and propranolol response. Additionally, exosome-mediated transfer of miRNAs between cells within the tumor microenvironment has emerged as an important mechanism of intercellular communication in IH. Understanding these regulatory networks may reveal novel therapeutic targets and biomarkers for disease monitoring (26, 27).

### 2.4 Cell-cell communication networks and disease progression

Chen et al. (14) performed scRNA-seq on proliferating-phase IH samples, identifying 10 major cell types and demonstrating that hemangioma mural cells constitute more than 50% of IH cells and promote endothelial proliferation through VEGFA and PGF paracrine signaling. The study further revealed that ADRB1 is selectively expressed in APLN-positive endothelial subpopulations, while ADRB2 is confined to other endothelial subsets, providing cellular resolution for β-receptor subtype-specific drug targeting. As disease progresses, cellular composition and communication networks undergo bidirectional remodeling, with pro-apoptotic and tissue remodeling programs progressively strengthening (14, 28). Ki-67-positive proliferating cells were primarily localized to mural cell clusters rather than endothelial clusters, a finding that extends beyond the traditional view that IH consists primarily of proliferating endothelial cells. Among endothelial subclusters, GLUT1-positive cells exhibited transcriptomic similarity to placental microvascular endothelial cells, consistent with the placental origin hypothesis. Collectively, these data suggest that the natural history of IH reflects coordinated interactions among endothelial cells, pericytes/mural cells, and the stromal microenvironment, rather than the behavior of any single cell type.

## 3 Mechanisms of IH phase transition: current hypotheses and unresolved questions

Although single-cell studies have revealed the cellular complexity of IH, the mechanisms governing the proliferation-to-involution transition remain incompletely understood. Understanding the cellular origins of IH is relevant to interpreting single-cell findings, as the identity of the founding progenitor determines the lineage composition observed in these atlases. Several origin hypotheses have been proposed, and single-cell data now offer a means of evaluating each.

### 3.1 Embryonic origin and stem/progenitor cell theory

The cellular origin of IH remains incompletely resolved, and several non-mutually exclusive hypotheses have been proposed (29). The placental origin theory is supported by shared marker expression (GLUT-1, Lewis Y antigen, FcγRII) between IH endothelial cells and placental microvascular endothelium, and by evidence that placental cell embolization during gestation may seed lesion formation (30, 31). The neural crest theory draws on the predominance of IH in the head and neck region and the expression of neural crest markers on IH endothelium (31–34). The stem/progenitor cell theory is supported by the identification of CD133-positive hemangioma-derived stem cells (HemSCs), which can differentiate into endothelial cells, pericytes, and adipocytes, and recapitulate IH in xenograft models (35). Single-cell analyses suggest that IH may originate from a dysregulated multipotent progenitor with hemogenic endothelial and neural crest features, consistent with both placental and neural crest origin theories (35–37). The discovery of CD146-positive HemMCs, which share mesenchymal stem cell properties, adds a further dimension to this picture: their adipogenic differentiation potential may account for the replacement of vascular tissue by fibrofatty stroma during involution (20).

### 3.2 Hypoxia and renin-angiotensin system-related mechanisms

Hypoxia is considered an important trigger for IH development. Local hypoxia can induce upregulation of hypoxia-inducible factor 1α (HIF-1α) expression, subsequently activating transcription of angiogenic factors including VEGF and promoting abnormal vessel formation (38). Additionally, the relationship between the renin-angiotensin system (RAS) and IH involution has attracted attention: physiological decline in plasma renin levels with age, or β-blocker-mediated inhibition of renin release, may drive the involution process (39). This suggests that endocrine-angiogenic networks may participate in regulating disease phase transition, although the key determining factors remain to be established.

## 4 Mechanisms of propranolol action: from classical effects to enantiomer-specific differences

### 4.1 Pharmacological basis and chiral structure

Propranolol is a non-selective β-adrenergic receptor blocker, with clinical formulations consisting of a 1:1 racemic mixture of R(+) and S(−) enantiomers (40). From a stereochemical perspective, the S(−) enantiomer possesses β-receptor blocking activity and is primarily responsible for cardiovascular effects (bradycardia, hypotension) and metabolic effects (hypoglycemia risk from glycogenolysis inhibition); in contrast, the R(+) enantiomer has minimal β-receptor blocking activity (41). This chiral difference provides important insights into understanding propranolol’s mechanism of action in IH treatment.

Infants have immature hepatic and renal metabolic function, limited glycogen reserves, and underdeveloped blood glucose regulatory capacity, rendering them particularly vulnerable to adverse effects of β-blockade including hypoglycemia, bradycardia, hypotension, and bronchospasm–though, as noted in Section 4.5, these serious events occur infrequently in clinical practice (8). Therefore, clarifying the contributions of different enantiomers has significant translational value–if the R(+) enantiomer can independently exert anti-IH effects, it may be possible to develop novel formulations that preserve efficacy while reducing these pharmacological risks.

### 4.2 Classical mechanisms of action

The therapeutic effects of propranolol in IH have been attributed to several mechanisms. β-receptor blockade reduces vascular smooth muscle responsiveness to catecholamines, decreasing lesion blood perfusion, which is thought to underlie the rapid lightening of lesion color observed within hours of drug administration (5, 42). Propranolol also downregulates proangiogenic factors including VEGF and basic fibroblast growth factor (bFGF) (43), inhibiting endothelial cell proliferation and tube formation (42). Additionally, propranolol induces IH endothelial cell apoptosis through the intrinsic apoptotic pathway, manifested by caspase-3 and caspase-9 activation and upregulation of Bax and p53 expression (43, 44). RAS system involvement is also considered an important component of propranolol-accelerated IH involution (39).

### 4.3 R(+) enantiomer and SOX18 pathway

Studies have shown that the two propranolol enantiomers differ in their effects on IH, providing new perspectives on its mechanism of action. Overman et al. (41), while studying hypotrichosis-lymphedema-telangiectasia and renal syndrome (HLTRS), discovered that R(+) propranolol functions as a small molecule inhibitor of the transcription factor SOX18, with this effect independent of β-receptor blocking activity.

SOX18 belongs to the SRY-related HMG-box transcription factor family and plays critical roles in vascular development and endothelial cell differentiation. Studies have demonstrated that R(+) propranolol interferes with formation of both SOX18 homodimers (SOX18:SOX18) and SOX18-RBPJ heterodimers (SOX18:RBPJ), impeding SOX18 binding to chromatin and thereby inhibiting target gene transcription (41). In the IH context, this mechanism has clear functional significance: R(+) propranolol effectively inhibits HemSC differentiation toward endothelial cells, with inhibitory effects comparable to the known SOX18 small molecule inhibitor Sm4 (41).

Seebauer et al. (45) further confirmed that both R(+) propranolol and R(+) atenolol inhibit HemSC-derived vessel formation in mouse xenograft models without affecting body weight or blood glucose levels. This study also found that R(+) propranolol significantly downregulates expression of the angiogenesis-related factor angiopoietin-like protein 4 (ANGPTL4) and activates tumor suppressor genes including betaine-homocysteine methyltransferase and early growth response factor 1 (46).

These findings have important translational implications. Approximately 10%–25% of IH patients respond poorly to propranolol or experience rebound after discontinuation (9, 47); the independent anti-angiogenic activity of the R(+) enantiomer provides a potential alternative therapeutic strategy for these patients. Development of pure R(+) enantiomer formulations could theoretically preserve anti-IH efficacy while avoiding S(−) enantiomer-mediated β-receptor blockade-related adverse effects (hypoglycemia, bradycardia, bronchospasm), which would be of significant value for infant medication safety.

Subsequent work has identified a SOX18-mevalonate pathway (MVP) axis in IH pathogenesis. Transcriptomic profiling of patient-derived HemSCs revealed that R(+) propranolol causes SOX18-mediated downregulation of MVP genes during endothelial differentiation. The MVP plays critical roles in cholesterol biosynthesis and has been increasingly recognized for its involvement in angiogenesis and vascular development. Functional validation in preclinical IH models demonstrated that statins–targeting the MVP–are potent inhibitors of hemangioma vessel formation. These findings suggest that statins may represent an alternative or adjunctive therapy for patients who respond poorly to β-blockers. Clinical trials investigating topical simvastatin formulations for IH are currently underway; their results will help determine whether this approach represents a viable additional treatment option for affected infants (48).

### 4.4 Cell-targeting mechanisms and interaction network regulation

Single-cell studies have also provided insight into which cell types propranolol acts upon and how these actions collectively promote involution.

At the endothelial cell level, propranolol dose-dependently inhibits hemangioma endothelial cell proliferation, migration, and tube formation (42), and downregulates molecules including EGFL7 to affect angiogenesis (42). Propranolol also upregulates adipogenesis-related genes including PPARγ in HemSCs, promoting their differentiation toward adipocytes and accelerating involution (44). From a developmental signaling perspective, research has found that propranolol can modulate Notch-related pathways in endothelial cells (such as the Jagged1/Notch axis), influencing endothelial cell fate determination and vascular maturation (49, 50). This is consistent with the adipogenic potential of CD146-positive HemMCs; propranolol may accelerate involution in part by promoting their differentiation toward adipocytes.

Based on the findings of Chen et al., propranolol may exert effects through inhibiting APLN-positive endothelial cell function and downregulating Apelin/APLNR signaling (14); post-treatment serum apelin levels decrease in synchrony with lesion regression (15). The proportion of CENPF-positive pericytes decreases significantly following propranolol treatment, suggesting that propranolol downregulates proliferation-related programs in this subpopulation (14, 19). These subpopulations and their associated signals are candidate indicators for monitoring treatment efficacy (14–16).

In summary, single-cell studies indicate that propranolol acts on several distinct cell populations within IH tissue. Propranolol suppresses APLN-positive endothelial subpopulations and attenuates Apelin/APLNR signaling; serum apelin levels decline with lesion regression and may serve as a marker of treatment response (14–16). The R(+) enantiomer inhibits SOX18, blocking HemSC differentiation toward endothelial cells and supporting the potential use of statins as adjunctive therapy through the SOX18–mevalonate pathway axis (41, 46). The proportion of CENPF-positive pericytes decreases following propranolol treatment, and propranolol promotes adipogenic differentiation of CD146-positive HemMCs (19, 20). Whether the Apelin/APLNR and SOX18 mechanisms interact at a molecular level has not been established; single-cell data indicate that they operate in distinct cellular compartments, with APLN expression concentrated in tip-like endothelial cells and SOX18 activity relevant at the HemSC stage (14) (Table 1).

**TABLE 1:** Key cellular subpopulations identified in IH and their responses to propranolol treatment.

Cell subpopulation	Disease phase	Key characteristics	Identification method	Propranolol response
APLN^+^ HemECs	Proliferation	IH-specific endothelial subtype with tip cell characteristics; drives pathological angiogenesis via Apelin/APLNR axis (14, 16)	scRNA-seq: identified as IH-specific subtype enriched in proliferating phase; significantly reduced after propranolol treatment (14)	Propranolol suppresses APLN/APJ axis activity; ADRB1 selectively expressed in APLN^+^ subpopulation; serum APLN correlates with treatment response (14–16)
CENPF^+^ Hem-pericytes	Proliferation	Highly proliferative perivascular cells; CENPF expression marks G2/M phase; Ki-67 co-staining confirms proliferative activity (14, 19)	scRNA-seq: identified as IH-specific pericyte subtype; absent in normal skin; proportion decreases with propranolol treatment (14)	Proliferative programs downregulated following propranolol treatment; proportion of CENPF^+^ pericytes decreases significantly (14, 19)
CD146^+^ mural cells (HemMCs)	Proliferation/involution	Constitute > 50% of IH cells; possess mesenchymal stem cell properties; exhibit proangiogenic-to-adipogenic differentiation switch (14, 20)	scRNA-seq: mural cells constitute > 50% of IH cells, with Ki-67^+^ proliferating cells localized to mural clusters; proangiogenic activation and adipogenic potential confirmed by CD146 sorting and transcriptomic analysis (14, 20)	Propranolol upregulates PPARγ and accelerates adipogenic differentiation; promotes fibrofatty replacement underlying spontaneous involution (20, 44)
HemSCs (CD133^+^)	Proliferation	Multipotent progenitor; differentiates into endothelial cells, pericytes, and adipocytes; recapitulates IH in xenograft models (35)	*In vitro* functional studies and xenograft model: Using anti-CD133 magnetic bead selection (35); subsequently identified as a distinct transcriptomic cluster by scRNA-seq (14)	R(+) propranolol: SOX18 inhibition blocks HemSC-to-endothelial differentiation; SOX18–mevalonate pathway axis supports statin repurposing potential (41, 46)
M2 macrophages	All phases	Predominant immune component in IH; promote endothelial differentiation and angiogenesis; inhibit HemSC adipogenesis (21)	Histology and immunophenotyping: M2 polarization confirmed by IHC and flow cytometry; M2-derived exosomes identified by functional studies (21, 22)	M2-derived exosomal miR-27a-3p reduces HemSC sensitivity to propranolol; immune microenvironment modulates drug response (22)
Mast cells	All phases	Release proangiogenic mediators (SCF, VEGF, bFGF) during proliferating phase; secretory profile shifts during involution, potentially facilitating fibrofatty replacement (23)	Histological staining and IHC: tissue distribution across IH phases characterized by light microscopy; no dedicated scRNA-seq dataset currently available (23)	Direct effect of propranolol on mast cells in IH not established; β-adrenergic receptors expressed on mast cells in other tissue contexts
Telocytes (AQP1^+^)	All phases	Specialized stromal cells forming perivascular networks with HemMCs, α-SMA^+^ cells, and CD31^+^ endothelial cells; AQP1 expression suggests role in local fluid homeostasis (25)	Immunofluorescence and confocal microscopy: AQP1-expressing TC layers identified surrounding IH vessels; spatial networks with CD146^+^ HemMCs, α-SMA^+^ and CD31^+^ endothelial cells characterized (25)	AQP1-dependent response to β-blockade demonstrated; spatial proximity to HemMCs suggests involvement in tissue remodeling during propranolol treatment (25)

APLN, apelin; APJ/APLNR, apelin receptor; AQP1, aquaporin-1; CENPF, centromere protein F; HemEC, hemangioma endothelial cell; HemMC, hemangioma mural cell; HemSC, hemangioma stem cell; IH, infantile hemangioma; IHC, immunohistochemistry; PPARγ, peroxisome proliferator-activated receptor gamma; SCF, stem cell factor; TC, telocyte.

### 4.5 Adverse effect mechanisms and risk management

The most commonly reported adverse effects of propranolol in clinical practice are mild and reversible: gastrointestinal disturbance, sleep disturbance, and acrocyanosis (6). Serious adverse effects related to S(−) enantiomer-mediated β-receptor blockade–including symptomatic hypoglycemia, bradycardia, hypotension, and bronchospasm–occur infrequently; in the pivotal RCT, these events were reported at similar frequencies in propranolol and placebo groups (6). Nevertheless, hypoglycemia risk arises from β-blockade-impaired gluconeogenesis and glycogenolysis, and risk is significantly elevated during infection with fever, diarrhea/vomiting, reduced feeding, or prolonged nocturnal fasting (8).

In practice, pre-treatment assessment should identify high-risk infants, including those born prematurely, with low birth weight, feeding difficulties, or a history of hypoglycemia or seizures. Vital signs and blood glucose should be monitored during initiation and dose escalation (4, 51). Parents should be informed of warning signs (feeding refusal, lethargy, sweating, pallor, seizures), and clinicians should remain alert to bronchospasm risk in infants with a history of wheezing or recurrent respiratory infections. Should R(+) enantiomer formulations enter clinical application in the future, these adverse effect risks could be reduced. The converse risk is also clinically relevant: β_2_-AR agonists (e.g., salbutamol, procaterol) administered concurrently or after propranolol discontinuation can acutely reactivate β-AR-driven proangiogenic pathways, triggering rapid IH re-proliferation or rebound. Case reports involving intravenous and nebulized β_2_-agonists confirm this risk and underscore the need to avoid β_2_-AR agonist exposure in IH patients whenever possible (52).

## 5 Treatment response heterogeneity, rebound mechanisms, and stratified therapy prospects

Clinical observations demonstrate significant individual variation in IH response to propranolol, with some patients responding poorly and others experiencing rebound following treatment discontinuation. Rebound rates of approximately 10%–25% (9, 52) are associated with lesion location, type, sex, and timing of discontinuation, although specific mechanisms remain unclear.

From a cellular standpoint, rebound may reflect the re-emergence of proangiogenic activity once pharmacological suppression is withdrawn. If residual cellular subpopulations with high angiogenic potential (such as APLN-positive endothelial cells or CD146-positive HemMCs that have not completed adipogenic differentiation) or persistent proangiogenic signals from immune/stromal microenvironment remain at treatment discontinuation, re-proliferation becomes more likely. Case reports of β-receptor agonist-induced acute IH deterioration (52) indirectly support the association between β-receptor-related pathways and lesion activity. Based on the R(+) enantiomer-SOX18 mechanism discovery, some patients with “poor propranolol response” may harbor driving mechanisms beyond SOX18 pathways, requiring further investigation.

Based on associations between cellular subpopulations and translatable biomarkers, a “baseline-early response-pre-discontinuation status” three-phase monitoring framework could be constructed. For example, enrichment of APLN-positive endothelial cells in proliferating lesions may correspond to more active angiogenic states, with elevated serum apelin reflecting systemic output from this pathway; post-treatment apelin decline may parallel clinical regression. Whether the degree of CD146-positive HemMC adipogenic differentiation correlates with rebound risk remains to be investigated; incomplete adipogenic transition could in principle leave residual proangiogenic activity at the time of treatment discontinuation. Prospective validation of these associations could support more evidence-based approaches to treatment monitoring and decisions about when to discontinue therapy.

## 6 Discussion

This review has examined the cellular heterogeneity of IH and the cellular basis of propranolol action, drawing on recent single-cell omics studies. Below we discuss key findings, areas of ongoing uncertainty, and directions for future research.

### 6.1 Fundamental concepts and emerging framework

Single-cell RNA sequencing has yielded high-resolution cellular maps of IH tissue, advancing our understanding of this condition. Current evidence suggests that IH disease progression may be driven by “dynamic switching of dominant cellular subpopulations” within multicellular networks, rather than simple endothelial cell hyperproliferation alone. The proliferating phase is dominated by proangiogenic subpopulations including APLN-positive endothelial cells and CENPF-positive pericytes; CD146-positive HemMCs show notable changes during the proliferation-to-involution transition, with their “proangiogenic-adipogenic” dual potential closely matching the natural history of IH; the involuting phase then shifts toward pro-apoptotic and tissue remodeling programs. This “subpopulation-centered” model differs from the traditional “endothelial cell-centric” view of IH pathogenesis.

Regarding propranolol, the emerging picture is one of enantiomer-specific and multi-cellular mechanisms rather than simple β-receptor blockade. SOX18 inhibition by the R(+) enantiomer provides a mechanistic rationale for developing formulations with reduced adverse effects, and the SOX18–mevalonate pathway axis raises the possibility that statins could serve as an adjunctive or alternative treatment in selected patients.

### 6.2 Controversies and different schools of thought

Several controversies remain in the IH field. First, regarding the cellular origin of IH, competing hypotheses include the placental origin theory (supported by shared marker profiles between IH endothelial cells and placental microvascular endothelial cells, including GLUT1 expression), the stem/progenitor cell theory (supported by the identification of multipotent HemSCs and CD146-positive HemMCs), and the hypoxia-driven vasculogenesis hypothesis (supported by elevated HIF-1α and VEGF expression in proliferating lesions). These are not necessarily mutually exclusive, and the relative contribution of each mechanism may vary among patients.

Second, the identity of the “key driver cell” of IH remains debated: while traditional views emphasize endothelial cell hyperproliferation, emerging single-cell data suggest that mural-like cells may constitute the predominant proliferating population, with CD146-positive HemMCs proposed as a cellular link between the proliferating and involuting phases through their bipotent differentiation capacity. This question has practical implications for which cell populations should be targeted therapeutically.

Third, the relative importance of β-receptor-dependent versus β-receptor-independent mechanisms in propranolol’s therapeutic effects remains to be definitively established. While the discovery of SOX18 inhibition by R(+) propranolol has illuminated a key β-receptor-independent pathway, the clinical contribution of this mechanism relative to classical β-blockade effects (vasoconstriction, anti-angiogenesis, pro-apoptosis) requires further clarification. Resolving this question would inform decisions about treatment formulation and the potential development of enantiomer-specific agents.

### 6.3 Current research gaps

Despite these advances, several interconnected gaps limit further progress. At the level of data resolution, existing scRNA-seq datasets lack spatial context: it remains unknown where APLN-positive endothelial cells and CD146-positive HemMCs are located relative to one another within lesions, and how this spatial organization shifts across disease stages. Spatial transcriptomics is the most pressing methodological need in the field.

More fundamentally, the molecular trigger of the proliferation-to-involution transition remains unresolved. Whether this switch is cell-autonomous, driven by microenvironmental signals, or governed by systemic factors such as age-related renin-angiotensin changes is unknown–and this question cannot be answered by cross-sectional single-cell data alone. Related to this, the relationship between the Apelin/APLNR axis and SOX18-mediated mechanisms has not been explored at a molecular level. Given that these pathways operate in distinct cellular compartments, determining whether they converge, act independently, or interact indirectly through paracrine networks would substantially clarify propranolol’s mechanism of action.

On the translational side, the field currently lacks validated biomarkers for staging or treatment monitoring. Serum apelin shows early promise, but prospective cohort data are absent. Earlier candidate serum biomarkers such as circulating VEGF, reported to distinguish proliferating from involuting IH and from vascular malformation (53), have not been adopted as routine staging markers, underscoring the need for validated, subpopulation-anchored biomarkers. Similarly, the mechanisms underlying the 10%–25% of patients who respond poorly or experience rebound remain poorly characterized; immune microenvironment factors–particularly M2 macrophage-derived exosomal miR-27a-3p–are plausible contributors, but mechanistic and clinical evidence is limited. The regulatory roles of ncRNAs and the long-term neurodevelopmental safety of early propranolol exposure are important questions, though arguably secondary to resolving the core mechanistic and biomarker gaps first.

### 6.4 Future directions and potential developments

Several directions merit priority. Integrating spatial transcriptomics with scRNA-seq would define the tissue localization of key subpopulations, particularly CD146-positive HemMCs, and how this changes across disease stages. Prospective validation of candidate biomarkers, such as serum apelin, is needed to establish their utility for monitoring treatment response, guiding treatment duration, and assessing rebound risk. Preclinical and clinical evaluation of R(+) propranolol is warranted to determine whether anti-IH efficacy can be preserved with fewer β-blockade-related adverse effects.

Further areas of investigation include statin repurposing via the SOX18–mevalonate axis, subpopulation-targeted approaches such as promoting HemMC adipogenic differentiation, and characterization of the ncRNA regulatory network as a potential source of therapeutic targets and biomarkers. The role of exosome-mediated intercellular signaling in modulating treatment response also warrants further study. Development of standardized criteria for disease staging and treatment response assessment would further support progress in this area.

Progress in these areas would support a shift toward more mechanism-informed management of IH, with the aim of improving outcomes and reducing treatment burden for affected infants.
